# Detecting progressing events in multiple sclerosis clinics in real-time is possible: Yes

**DOI:** 10.1177/13524585251409568

**Published:** 2026-01-14

**Authors:** Chao Zhu, Helmut Butzkueven

**Affiliations:** Department of Neuroscience, School of Translational Medicine, Monash University, Melbourne, VIC, Australia; Department of Neuroscience, School of Translational Medicine, Monash University, Melbourne, VIC, Australia

## Introduction

The Pathophysiology of disease progression in multiple sclerosis (MS) is complex, involving a variable mix of axonal and neural degeneration, progressive demyelination, and acute and chronic central nervous system inflammation. The gradual nature of progression requires repeated structured assessments in the MS clinic to enable real-time detection.

## Clinical assessment of disability progression

The Expanded Disability Status Scale (EDSS) scale^1^ underpins assessment of MS disease severity. The EDSS is appealing because it is fundamentally based on the findings of the neurological examination, plus walking capacity. Despite frequent criticisms, it has stood the test of time. The EDSS scale has evolved into the highly standardised Neurostatus EDSS which includes a certification process for clinicians. Currently, the term ‘progression event’ is largely synonymous with EDSS progression (i.e. worsening). The most common definition sets a threshold for increase dependent on baseline EDSS. This increase can be confirmed with a follow-up EDSS score, either 3,6 or 12 months later, then termed ‘Confirmed Disability Progression’ (CDP). Finally, progression events can be categorised as relapse associated worsening (RAW) or progression independent of relapse (PIRA).^2^ Provided an MS clinician assesses and records EDSS scores in the clinic, real-time detection of EDSS progression, CDP, PIRA and RAW events is, of course, possible. Ideally, EDSS time series data is available to the clinician in a graphical interface to further simplify real-time detection of progression. It is also an important clinical standard, because progression is a potential indicator of suboptimal treatment response and should trigger a treatment change discussion.

## Is detection of EDSS progression feasible in routine clinical care? YES!

Hundreds of registry-associated clinics in Denmark, Sweden, France, Italy, and clinics from over 40 countries in the MSBase Registry all perform regular EDSS scores, as do many other MS centres. The registry-associated clinics follow over 330,000 MS patients.^3^ As an example, the MSBase registry contains over 1,000,000 EDSS scores recorded during clinical care. These registry datasets generate much of the current research characterising PIRA and RAW events. This includes assessments of optimal PIRA definitions,^4^ frequency and determinants of CDP in MS populations,^5^ long-term PIRA persistence^6^ and PIRA/RAW prognostic significance.^7^ However, whether EDSS scoring is possible in clinics depends first on time allocations for standard clinic reviews, which range from 5 minutes (South Korea, Taiwan) to 30+ minutes (Australia, Italy, France, Sweden, Denmark), and second on staff motivation. Incorporating EDSS scoring as a clinical quality indicator could increase motivation, but there are only few examples of quality care standards in MS at present. Tele-EDSS or self-assessed EDSS scores are also validated and used in settings with a lot of tele-health appointments or clinician resource limitations.^8^

## Non-EDSS measures of progression

Other clinical assessments of progression use tests included in the MS Functional Composite Score (MSFC), currently consisting of the timed 25-foot walk test, the nine-hole peg test to quantitate upper limb function and the Symbol Digit Modality test (SDMT) to assess cognitive function. Confirmation of progression on either the composite score or its individual components uses analogous definitions to that used for EDSS-based CDP. Given these tests require clinical time to administer, they are mostly used in clinical trial settings, with some notable exceptions (e.g. the SDMT is widely used in routine care in Sweden).

One possible future direction is ‘APPification’ of EDSS, MSFC-related and many other functional tests that people with MS could perform using personal smartphones or tablets.^9,10^ While home-based testing persistence is a major issue, they could certainly be performed at clinic visits in a waiting room area. Although some additional space would be required, it would cost little or no extra staff time.

## Conclusion

Disability progression in MS is important to detect in routine quality clinical care in real time, as it has major treatment implications. If serial EDSS scores are performed and accessible in the clinic, this is highly achievable. In the future, we believe smartphone-based self-assessment tools will increasingly be used and validated. These could possibly take over from the EDSS in the next few years, as they can provide a more granular and continuous measure of various neurological functions without the need for additional staff time in the clinic setting.
